# 

**Published:** 1845-02

**Authors:** 


*Our Exchauges.*—The *Medical News* commenced a new volume with the New Year. This publication, besides a mass of general intelligence interesting to the profession, has presented to its subscribers, in the last two volumes, the complete lectures of Dr. Watson, a work of no ordinary value. In the present volume commences the re-publication of Lectures on Surgery, delivered at St. George's Hospital, by *Sir Benjamin Brodie Bart*, F. R. S. &c. Our recommendation can add nothing to the reputation of this work. The Medical news is issued gratuitously to the subscribers to the American Journal of the Medical Sciences, published by Lea & Blanchard, Philadelphia. The subscription to the *News* alone is but $1 a year.

*The Medical Examiner*, appears in the volume for 1845 in a new form. During the year 1844 it was issured once a fortnight, each number covering 12 pages large octavo. In the new series it appears monthly, but much enlarged, presenting its subscribers with 72 instead of 24 pages, *and for the same money*. The subscription price continues to be $3; Dr. Robert M. Huston continues to conduct the Examiner, which alone will recommend it to all former subscribers. Lindsay & Blackiston, Philadelphia, are the publishers. ED.

